# Direct Granule Feeding of Thermal Droplet Deposition 3D Printing of Porous Pharmaceutical Solid Dosage Forms Free of Plasticisers

**DOI:** 10.1007/s11095-022-03198-x

**Published:** 2022-02-22

**Authors:** Thomas McDonagh, Peter Belton, Sheng Qi

**Affiliations:** 1 School of Pharmacy, University of East Anglia, Norwich, UK; 2 School of Chemistry, University of East Anglia, Norwich, UK

**Keywords:** Fused deposition modelling (FDM), Arburg Plastic Freeforming (APF), Thermal droplet deposition 3D printing, Porous solids, Solid dosage forms

## Abstract

**Purpose:**

To develop a new direct granule fed 3D printing method for manufacturing pharmaceutical solid dosage forms with porous structures using a thermal droplet deposition technology.

**Methods:**

Eudragit® E PO was used as the model polymer, which is well-known to be not FDM printable without additives. Wet granulation was used to produce drug loaded granules as the feedstock. The flow and feedability of the granules were evaluated. The physicochemical properties and in vitro drug release performance of the granules and the printed tablets were fully characterised.

**Results:**

Using the method developed by this study, Eudragit E PO was printed with a model drug into tablets with infills ranging from 30–100%, without additives. The drug was confirmed to be molecularly dispersed in the printed tablets. The printing quality and performances of the porous tablets were confirmed to be highly compliant with the pharmacopeia requirement. The level of infill density of the porous tablets had a significant effect on their in vitro drug release performance.

**Conclusion:**

This is the first report of thermal droplet deposition printing via direct granule feeding. The results of this study demonstrated that this new printing method can be used as a potentially valuable alternative for decentralised pharmaceutical solid dosage form manufacturing.

## Introduction

Additive manufacturing (AM) is a rapidly growing field encompassing many 3D printing technologies that have the potential to revolutionise the medical and pharmaceutical sector. Whilst some AM technologies such as binder jetting and selective laser melting are already used in high cost, low volume applications such as prosthetic and dental implant manufacture [1–5], only in the past few years have lower cost, high volume applications such as pharmaceutical drug delivery become viable [6–10]. Despite the huge increase in accessibility to 3D printers and large reductions in costs there is still only one 3D printed oral dosage form on the market, Spritam™ which gained FDA approval in 2015 [11]. The advantages of using 3D printing over traditionally mass produced ‘one-size fits all’ conventional solid dosage forms have been widely reported with flexible AM techniques enabling on-demand manufacture of personalised medicine tailored for individual patients’ clinical needs in terms of both dose and drug release profile [12, 13].

Within this research space, a range of 3D printing methods have been reported, including fused deposition modelling (FDM), selective laser sintering (SLS), binder jetting (BJ), semi-solid extrusion (SSE) and stereolithography (SLA) [13]. For melt extrusion-based 3D printing, the most commonly quoted limitation of the technique is the lack of suitable polymers for FDM printing [12, 14–16]. For successful printing filaments must possess mechanical properties, flexibilities and melt viscosities within a narrow range [17–19]. However, most pharmaceutical grade polymers lack such attributes, being either too brittle so that the filaments break in the motor gear or too soft to be pushed by the drive gear hindering printing [20, 21]. This limitation is even more apparent for immediate release polymers which count for approximately 70% of all oral dosage forms [22]. In order to make some filaments have suitable mechanical properties for printing, it is common to incorporate large quantities of additives and plasticisers into the formulation. For example, Sadia et al. [23] used immiscible fillers, talc, triethyl citrate (TEC) and tri-calcium phosphate (TCP) at 50:50 ratio with Eudragit EPO. Lactose, microcrystalline cellulose, magnesium stearate, tween 80 are other commonly used excipients [23–26]. In some studies plasticisers are incorporated for thermal reasons, lowering the HME and printing temperature of the formulation allowing the incorporation of more thermolabile API [10]. However, often these additional excipients for optimising the mechanical properties of the filaments do not add any functionality to the main drug delivery and absorption functions, leading to the design of complex formulations with increased tablet weight and increased potential for the adverse stability of the pharmaceutical product.

Therefore, research efforts have been dedicated to modifying the hardware of the printer to bypass the filament making and feeding. This will not only avoid the feeding issue, but also thermally process the excipients and APIs only once. In contrast, using filament-fed FDM, the API would be first thermally processed by HME, then thermally printed by FDM. This significantly increases the risk of thermally induced degradation. Modified FDM with power feeding has been reported [27, 28]. However, the feed mechanism is often not sufficient to process polymers with high melt viscosities and there can be the practical drawback associated with the powder flow to ensure continuous feeding.

**Fig. 1:**
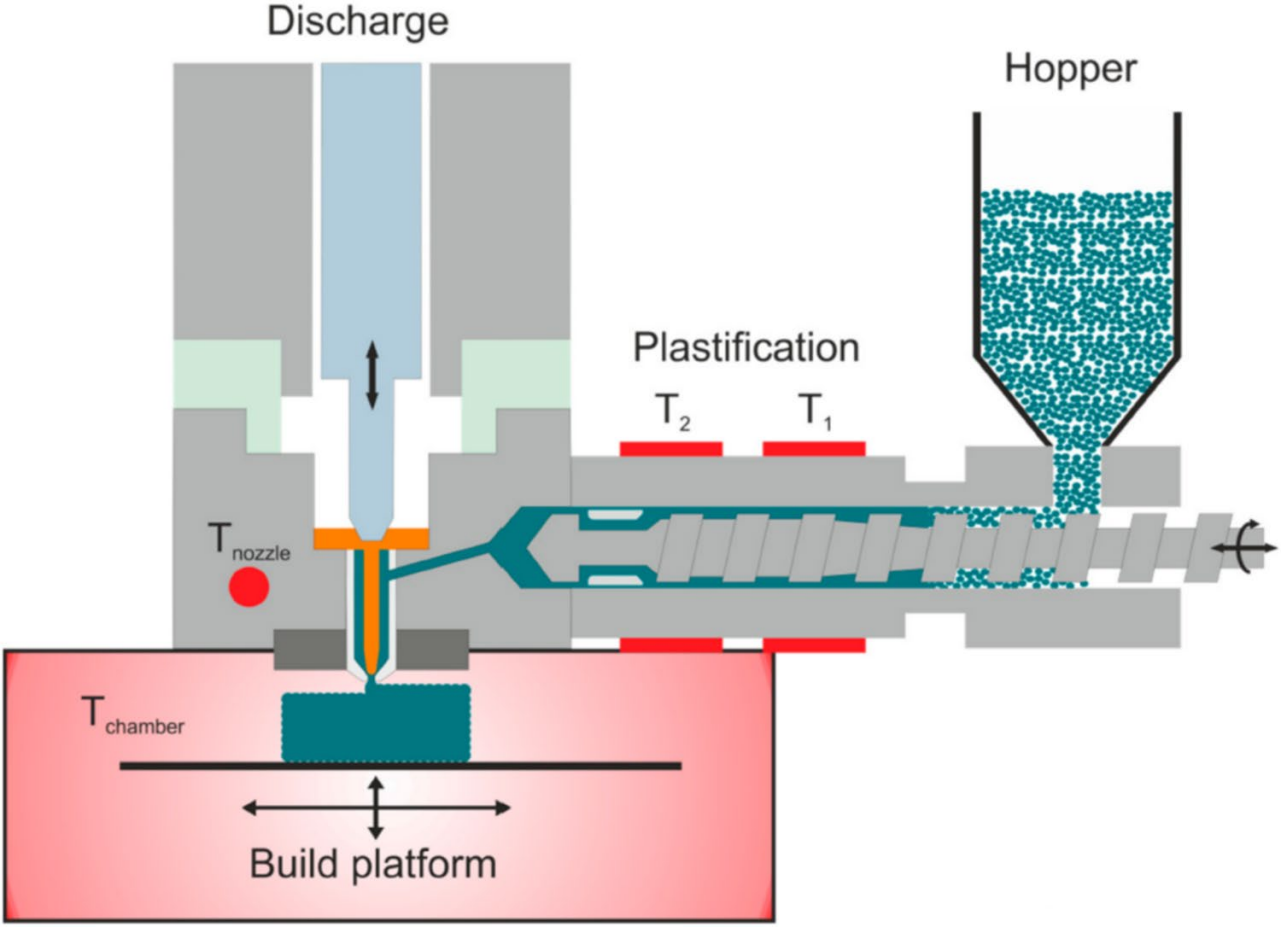
Graphic illustration of operational principle of APF printing. Raw materials in the form of pellets/granules are fed from the hopper. Single screw extruder with cylinder zone 1 (T_1_) and zone 2 (T_2_) which can be independently controlled to heat material. The molten material from Zone 2 is pushed into the print head at temperature T_nozzle_. The nozzle pin oscillates vertically at a set frequency to discharge the printed material. (adapted from [32])

In this study we evaluated an alternative 3D printing method, a newly introduced thermal droplet deposition 3D printing, commercially known as Arburg plastic freeforming (APF) [29, 30]. Similar to FDM, APF is also a thermal 3D printing technology. However, APF is fed by thermoplastic pellets rather than a filament. Pellets are fed into a screw similar to injection moulding which heats the material gradually to the desired temperature and drives it to the injection nozzle where it is extruded as individual droplets using a high-frequency piezo actuated nozzle pin [31, 32]. The operational principle is illustrated in Fig. 1. The pellets are gradually heated until they reach peak temperature at the nozzle, relying on both temperature and the mechanical energy from the screw to fuse the pellets. Conventional use of APF requires the use of pellets within 2–3.2 mm diameter. However, for pharmaceutical use, such pellets would require hot melt extrusion and pelletisation prior to the APF printing. To avoid the twice thermal processing of the API and the formulation, we investigated the feasibility of using direct granule feeding for APF printing. The granules were produced using the widely known wet granulation method. In order to explore the limit of printability, we kept the ingredients in the formulation to the minimum: API and the key polymeric carrier, and no other additives such as plasticizers. The granule feed and high-pressure material processing means that material selection for APF is not as constrained as it is for FDM with the capacity to print brittle materials with high melt viscosities that would break up or clog FDM extrusion heads. We used Eudragit® EPO with paracetamol as a model system. Eudragit® E PO has been widely used in pharmaceutical hot melt extrusion (HME) indicating its good thermostability and extrudability, however it is not FDM printable on its own due to the brittleness of the filament and its high melt viscosity [33]. To our knowledge this is the first time Eudragit® EPO based dosage forms have been printed without additional excipients. We use this novel formulation to explore the feasibility of printing personalised tablets with tuneable release rates and assess the quality attributes of the printing technique against pharmacopeia requirements for traditionally produced solid dosage forms.

## Materials and Methods

### Materials

Eudragit® E PO was kindly donated by Evonik (Evonik Industries, Germany) and the model drug, paracetamol (PAC), was purchased from Molekula (Molekula Ltd, United Kingdom).

### Granulation preparation

Drug loaded granules were prepared with a binary mixture of Eudragit® E PO and paracetamol (model drug) at a weight ratio of 10:1 (w/w). The abbreviation of Eudragit-PAC is used throughout the paper for the Eudragit® E PO and paracetamol formulation with this fixed ratio. All materials were accurately weighed and pre-mixed in a Kenwood food mixer with flat beater attachment for 5 min. To agglomerate the powder into granules 50% v/w Milli-Q water was added and the formulations were further mixed for 15 min. Wet granules were subsequently removed from the food mixer, spread thinly over baking trays and placed in an oven set to 40 °C overnight. Once dried, granules would fall apart on light handling. A 850 μm and 2 mm sieve were used to collect granules of more uniform size distribution to improve flowability. These three sets of granules were collected.

### Flow property characterisation of the granules

Angle of repose and tapped density were used as the key methods to measure the flow properties of the granules. For measuring the angle of repose, granules were passed through a glass funnel positioned 15 cm above a desktop to view the steepest angle at which the material piled up. Tapped density measurements were taken on the 3 different granule sizes in triplicate. For each test, a quantity of granules (60–100 mL) was carefully poured into a graduated 100 mL volumetric cylinder without compacting. The unsettled apparent powder volume, V_0_, was measured to the nearest unit and the weight, m, of the powder was determined. The bulk density, ρ_0_, was calculated from the formula m/V_0_. To obtain the tapped density, ρ_tap_, a suitable mechanical tapped density tester that provides a fixed drop of 14 ± 2 mm at a nominal rate of 300 drops per minute was used conforming to USP method 1. The Hausner ratio is the ratio of ρ_tap_, ρ_0_.

### APF printing of tablets *via* direct granule feeding

An Arburg Freeformer equipped with 2 piezo actuated thermal extrusion heads (200 μm nozzle diameter) was used for printing the drug loaded tablets. Digital CAD files for the 3D geometries were generated in Solidworks and exported to Arburg’s proprietary slicing software (Freeformer software V2.2, Arburg, Germany) as an STL file. Tablets were printed onto a vacuum held Teflon build plate. Oval tablets were designed and printed with dimensions 15 mm in length, 7 mm maximum width and 3 mm height at infill densities from 30 to 100%. Printing conditions (shown in Table 1) were established using the material qualification procedure detailed in the results and discussion section.

**Table 1:** APF printing conditions for Eudragit® E PO with Paracetamol (10:1 w/w)

Feed material	Nozzle diameter (µm)	Temperatures (°C)			Build chamber temperature (°C)	Discharge (%)	Printing Pressure (bar)	Aspect Ratio (AR)	Layer height (mm)
		Zone 1	Zone 2	Discharge nozzle					
Eudragit-PAC granules	200	140	150	155	45	100	400–550	1.75	0.2

*Zone 1 is close to the feeding hooper and zone 2 is close to the discharge nozzle

### Scanning Electron Microscopy (SEM)

SEM was used to study the size and surface morphology of granules. All samples were attached to SEM stubs using double adhesive tape and then coated with gold using a Polaron SC7640 sputter gold coater (Quorum Technologies, Lauphton, UK) prior to imaging.

### Thermogravimetric analysis (TGA)

TGA was used to assess the thermal stability of the raw materials to ensure formulations were processed below the degradation temperature and to test the moisture content of granules produced via the wet granulation method. 5 – 10 mg of sample was loaded into titanium pans and subjected to a temperature program from 25—200 °C at 10 °C/min. Percentage weight loss at 105 °C was recorded as an indication of the water content present in the dried granules.

### Differential Scanning Calorimetry (DSC)

DSC was conducted using a Q20 differential scanning calorimeter (TA Instruments, Newcastle, United States) to detect glass transition temperatures (T_g_) and melting peaks for the different formulations. Raw materials, physical blends, granules and printed samples were characterised using a heat ramp with a temperature range of 0 °C to 200 °C at 10 °C/min with 1 min isothermal at 0 °C. Sample weights were 2–5 mg contained in an aluminium standard TA crimped pans and lids (TA Instruments, Newcastle, USA). All tests were done in triplicate.

### Powder X-ray diffraction (PXRD)

A D5005 X-ray diffractometer (Siemens, Munich, Germany) with monochromatic CuKα radiation (wavelength = 1.54056 Å) was used to measure the raw materials, the granules and the APF printed tablets. The extrudates and the printed tablets were briefly ground to powder form prior to their measurements. A scanning range of 5° < 2θ < 60°, with a step width of 0.02° and a scan speed of 4°/minute was used to conduct all measurements.

### Attenuated total reflectance Fourier transform infrared spectroscopy (ATR-FTIR)

ATR-FTIR measurements were conducted using a Vertex 70 FTIR spectrometer (Bruker Optics Ltd., United Kingdom), equipped with a Golden Gate, heat-enabled Attenuated Total Reflectance (ATR) accessory (Specac Ltd., Orpington, United Kingdom) fitted with a diamond internal reflection element. ATR-FTIR spectra were acquired in absorbance mode, using a resolution of 4 cm^−1^, 128 scans for each sample, within the range of wavenumbers from 4000 cm^−1^ to 600 cm^−1^. Spectra analysis was conducted using OPUS version 7.8 (Bruker Optics Ltd., United Kingdom). All measurements were done in triplicate.

### Drug content measurement

Accurately weighed drug loaded printed material was dissolved in a beaker containing 150 ml of 0.1 M pH 1.2 HCl (n = 6). The medium was stirred using a magnetic stirrer at room temperature until complete dissolution of all material. From this solution, 1 ml samples were withdrawn and filtered through a membrane filter with 0.45 μm pore size (Minisart NML single use syringe, Sartorius, UK). The drug concentrations of the samples were analysed using a UV–VIS spectrophotometer (Perkin-Elmer lambda 35, USA) at 243 nm using a UV cell with 1 cm pathlength. The drug content measurements were carried out in triplicate.

### *In vitro* drug release studies

The in vitro drug release profiles were measured in dissolution testing apparatus (Caleva 8ST, Germany) using the basket method (USP apparatus 1). A basket rotation speed of 100 rpm and 900 ml of pH 1.2 HCl at 37 ± 0.5 °C were used for all measurements. At each pre-determined time interval, 5 ml dissolution samples were withdrawn. The samples were directly filtered through a membrane filter with 0.45 μm pore size (Minisart NML single use syringe, Sartorius, UK). The samples were analysed using a UV–VIS spectrophotometer (Perkin-Elmer lambda 35, USA) at 243 nm. All dissolution tests were performed under sink conditions. All drug release studies were conducted in triplicate.

### *In vitro* drug release data analysis

Mean Dissolution time (MDT) was used to compare the release rates of the different tablet designs. MDT is a model independent parameter that allows the direct comparison of drug release rates of dosage forms [34–37]. The MDT is the time at which 50% of the drug is dissolved from its solid state under dissolution conditions. Such mathematical analyses enable statistical comparisons between the various formulations.

1 $$MDT=\frac{{\sum }_{j=1}^{n}{t}_{j*} \Delta {M}_{j*}}{{\sum }_{j=1}^{n} \Delta {M}_{j*}}$$

where j is the sample number, n is the number of dissolution sample times, t_j*_ is the time at a midpoint between t_j_ and t_j-1_ and ∆M_j*_ is the additional amount of drug dissolved between t_j_ and t_j-1_.

## Results and Discussion

### Granulation and granule characterisation

**Fig. 2:**
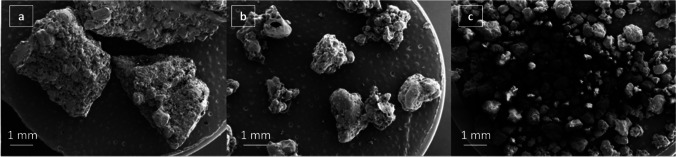
SEM images of (**a**) large; (**b**) medium and (**c**) small Eudragit-PAC granules produced via wet granulation

A typical wet granulation process with water as the granulating fluid was employed in this study. Wet granules of roughly the desired size (~ 1-3 mm) were dried at 40 °C. This is below the glass transition temperature of Eudragit® E PO, in order to prevent the granule softening and fusing. Using a sieving method, granules with three diameter ranges were produced, larger than 2 mm, between 850 μm and 2 mm and smaller than 850 μm. The medium size (between 850 μm and 2 mm) granules were used for printing as this population had the most narrow and well controlled particle size distribution. The images in Fig. 2 show that the granules produced are irregular in shape with rough surfaces. Weight loss of the dried granules was below 0.5% at 105 °C indicating that an overnight dry at 40 °C was sufficient for solvent removal.

The flow properties of the granules play a significant role in ensuring continuous feeding during APF printing. A high packing density with a low angle of repose is desirable for efficient loading and for smooth material flow into the screw for melting and printing [38].Therefore, tapped density and angle of repose measurements were performed. The packing density was found to improve with decreased granule size. This could be due to an increase in the particle size distribution in the smaller granules which enables the smaller granules to fill the gaps between the larger ones. The angle of reposes of the large, medium and small Eudragit-PAC granules are 44, 30, and 31.5°, respectively, indicating the large granules may have risk of needing flow aid during feeding, whereas the granules with medium and small sizes exhibited good flow properties [39], The calculated Hausner ratios are 1.08, 1.08, and 1.15 for the large, medium and small Eudragit-PAC granules, respectively. In practice all granule sizes were found to be printable. However, the large granules became logged in the neck of the hopper more frequently, preventing continuous material feed to the screw. This is in agreement with the flowability study that suggested the larger granules exhibited poorer flowability and could also be attributed to their higher relative size in comparison to the feeding tube. Therefore, considering their higher yield and better flow characteristics, the medium sized granules were used for further printing.

### Material qualification and APF printing optimisation

Material qualification is a term used by the printer manufacturer for a process required when printing new materials that are not in Arburg’s approved material database. During material qualification, parameters are tuned to obtain a homogenous melt, desired print resolution, optimum print density and layer adhesion without any material degradation. The material qualification process of Eudragit-PAC granules was performed, and the process is described below in 3 stages.

#### Stage 1 – Temperature adjustment

Using HME processing conditions as a guide temperature, material is discharged from the nozzle. Unlike FDM printers the temperature of 2 zones in the screw of the single screw extruder and the heated nozzle can be controlled to improve the melt homogeneity [32]. Increasing temperatures typically lowers the viscosity of the melt. Temperature is adjusted until a uniform flow of material discharges from the nozzle falling in circles approximately 5 cm in diameter.

The discharged filament should subsequently be viewed under a microscope to assess the filament quality and pick up issues related to moisture uptake or thermal/pressure degradation which can manifest as black defects or bubbles within the discharged filament. The temperatures settled on in this step act as a guide and are often further refined to optimise build plate/layer adhesion and to keep printing pressures in an acceptable pressure range (< 500 bar). Typically, the nozzle temperature is similar to that used with FDM printing with the zonal heating used to gradually get the material up to temperature, improving melt homogeneity.

#### Stage 2 – Determining drop aspect ratio (AR)

**Fig. 3:**
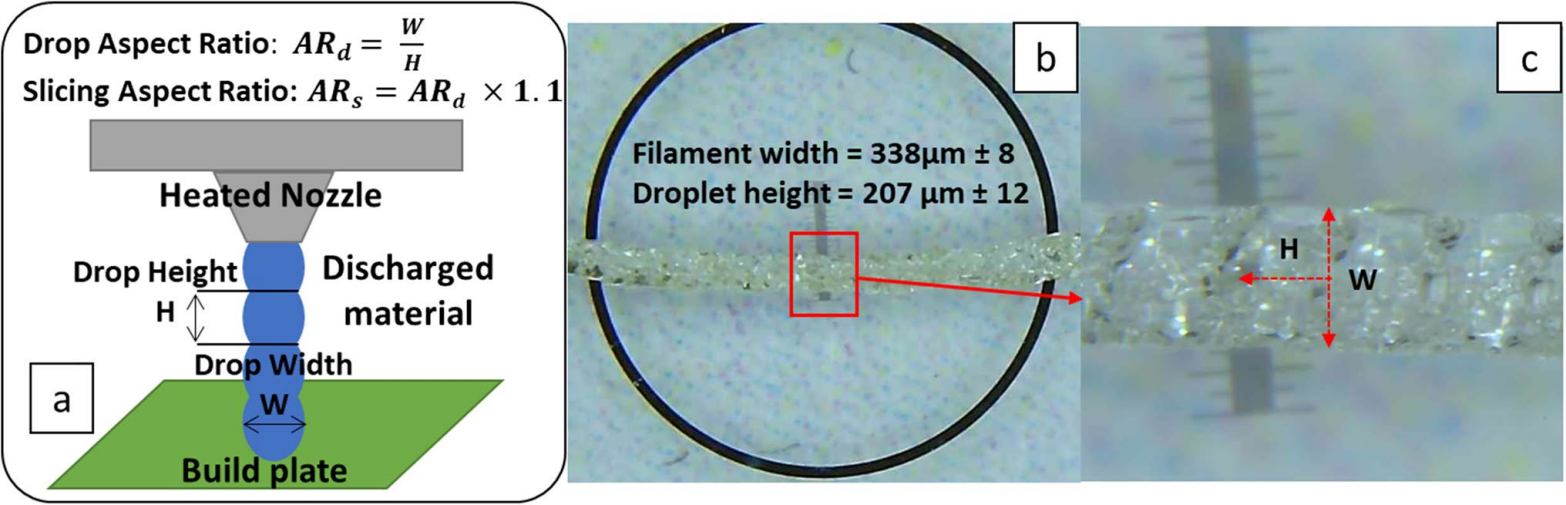
Graphic illustration of droplet aspect ratio experimental determination. H stands for droplet height and W stands for drop width; Droplet dimensions are measured from viewing discharged filament under a microscope. The slicing aspect ratio (AR_s_) inputted to the slicing software should be approximately 10% larger than the drop aspect ratio (AR_d_) to compensate for the merging of droplets, thus the AR_s_ is 1.1 times of AR_d_

Unlike FDM printers, material is deposited as droplets during APF printing rather than printpaths (also referred as ‘roads’ in some additive manufacturing literature). The volume of material deposited in each drop requires optimising to obtain prints with optimal part density. The deposited droplet geometries are material specific and vary based on material viscosity, temperature, and an APF parameter, discharge %, which controls the volume of material deposited in each drop. Generally, the discharge % parameter is varied to tune droplet volume to obtain the desired layer height. Drop aspect ratio (AR_d_) defines the spacing between adjacent drops. It can be estimated by measuring the length and width of discharged material as illustrated in Fig. 3. The slicing aspect ratio (AR_s_) inputted to the slicing software, should be approximately 10% larger than the drop aspect ratio (AR_d_) to compensate for the merging of droplets which occurs during printing. This compensation factor minimises the occurrence of voids between drops and improves droplet–droplet cohesion which results in greater print density and strength. For example, for the formulation used in this study a 200 μm LH was chosen so a droplet height of approximately 220 μm was targeted. A discharge of 100% gave a droplet height of 207 μm and a corresponding droplet width of 338 μm giving a drop AR_d_ of 1.63 and a slicing AR_s_ of 1.80.

#### Stage 3 – Refining drop aspect ratio

**Fig. 4:**
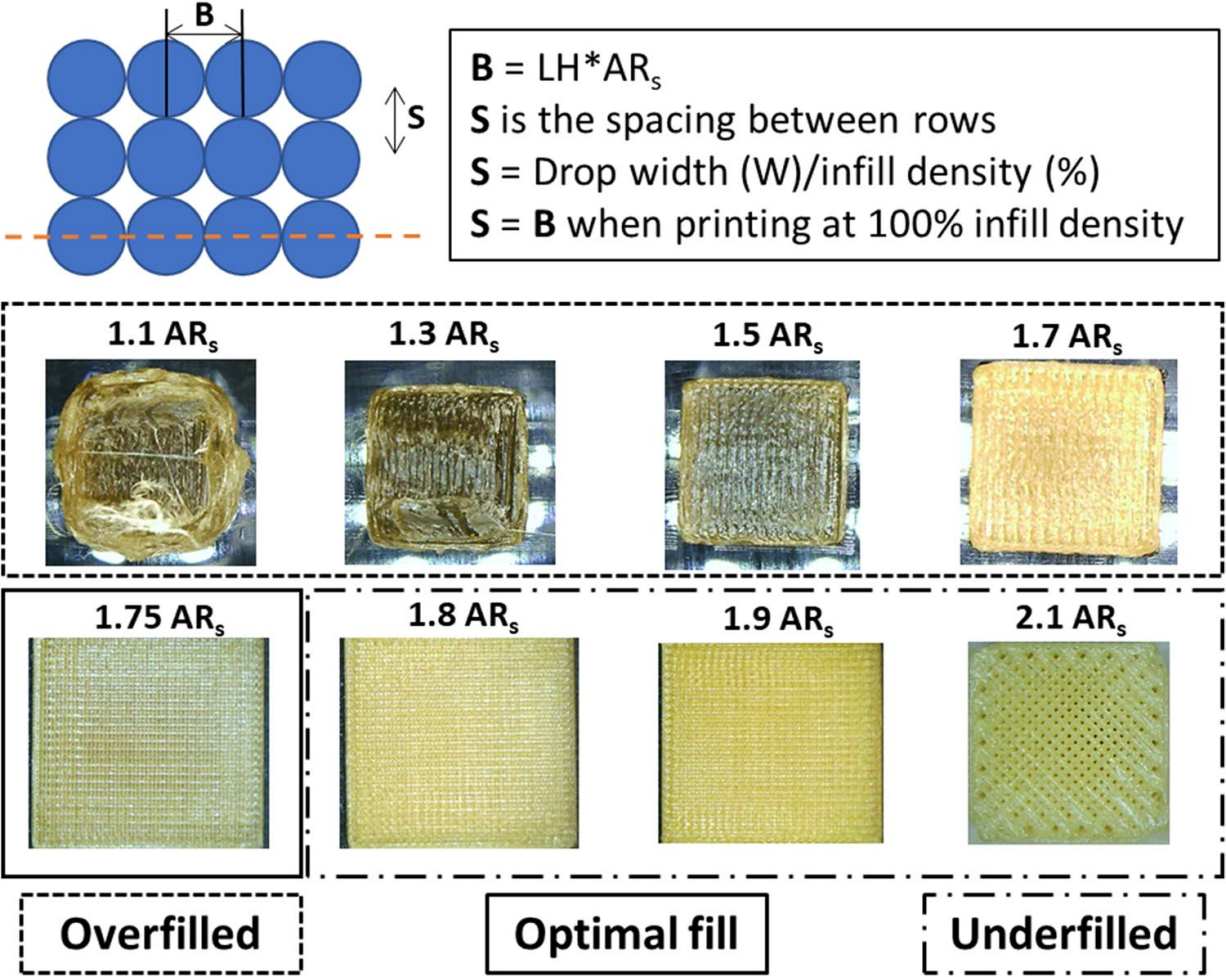
Graphic illustration of slicing aspect ratio (AR_s_) refinement to achieve optimum part filling, and surface finish; If AR_s_ is too low droplets will be deposited too close together resulting in an overfilled part and if AR_s_ is too high droplets will be too spaced out resulting in an underfilled part

The target of the AR optimisation is to achieve optimum part density and surface finish. Following the previous two stages, a set of parameters are set that act as a good baseline to begin printing optimisation. Using the AR value calculated from stage -2 a range of 20 mm cubes at 100% infill density were printed varying the aspect ratio in steps of 0.05 around our theoretical calculation. The surface finish of each print is then inspected. If the print is overfilled (Aspect ratio too low) the surface will appear wavy and if the print is underfilled small gaps between hatch segments will become visible. This process is shown in Fig. 4 for our formulation using an exaggerated range of aspect ratios from 1.1 to 2.1 with an AR of 1.75 found to give the optimal fill.

### APF printed porous tablets characterisation

**Fig. 5:**
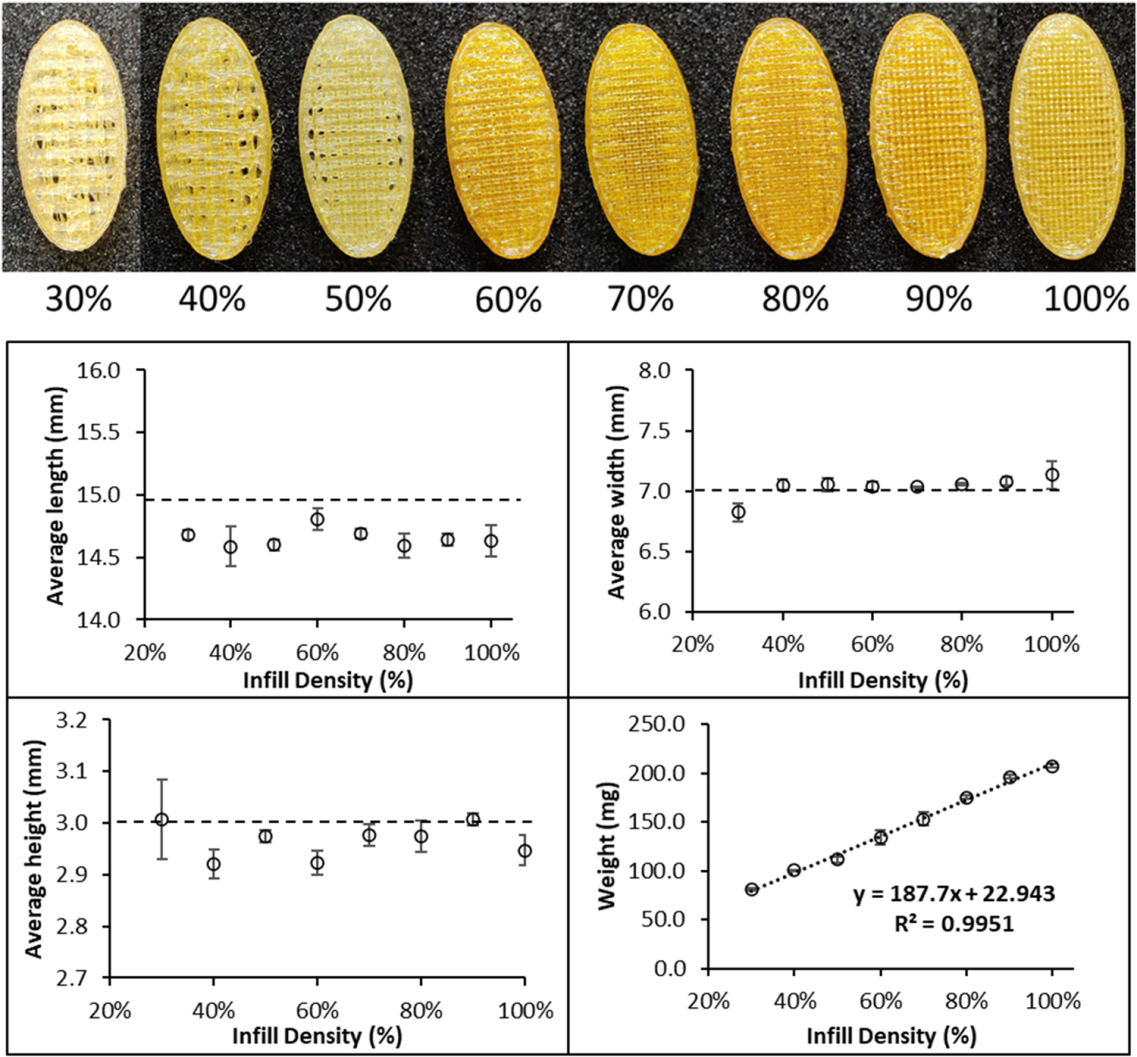
Visualisation of APF printed tablets with infills ranging from 30–100% (top); macroscopic dimension (length, width, height) and weight measurements of the tablets (bottom)

Eudragit-PAC granules were found to be suitable for APF 3D printing without the need to include other pharmaceutical excipients. Printing time was similar to that of FDM printing taking approximately 2 min per tablet. The large print bed 234 × 134 mm allows multiple tablets to be grouped together in a single print job making for efficient small batch production. All tablets showed good adhesion between the printed layers and little visible defects (Fig. 5). As seen in Fig. 5, the oval-shaped tablets with 30–100% infills show good shape fidelity in comparison to the target design dimensions. The tablet widths (widest measure across the middle of a tablet) show the closest match to the CAD design. The table height and length values deviated from the CAD design by less than 3% and less than 2%, respectively indicating a small amount of shrinkage, which is an-isotropic in the y-direction. Shrinkage can be corrected for using a scaling factor in the slicing software but due to being minimal was not used in this study. The tablet weights show excellent linear correlation with the infill density indicated by a R^2^ value of 0.995. For each infill, the variations between different prints are extremely low (as indicated by the SD values). Although there is no current pharmacopeia test for mass or content uniformity for small batch personalised medicine, the APF printing accuracy and reliability can be compared to the pharmacopeia tests for batch produced tablets. The weight uniformity specification has an upper limit of 85–115% of the average mass [40]. For sets of APF printed tablets printed (n = 6) with eight different infill densities, all individual tablets were within 93–105% of the average mass at each infill density. FDM printed tablet weight variabilities are commonly much larger due to the variable diameter of the feeding filaments which is difficult to control [41].

**Fig. 6:**
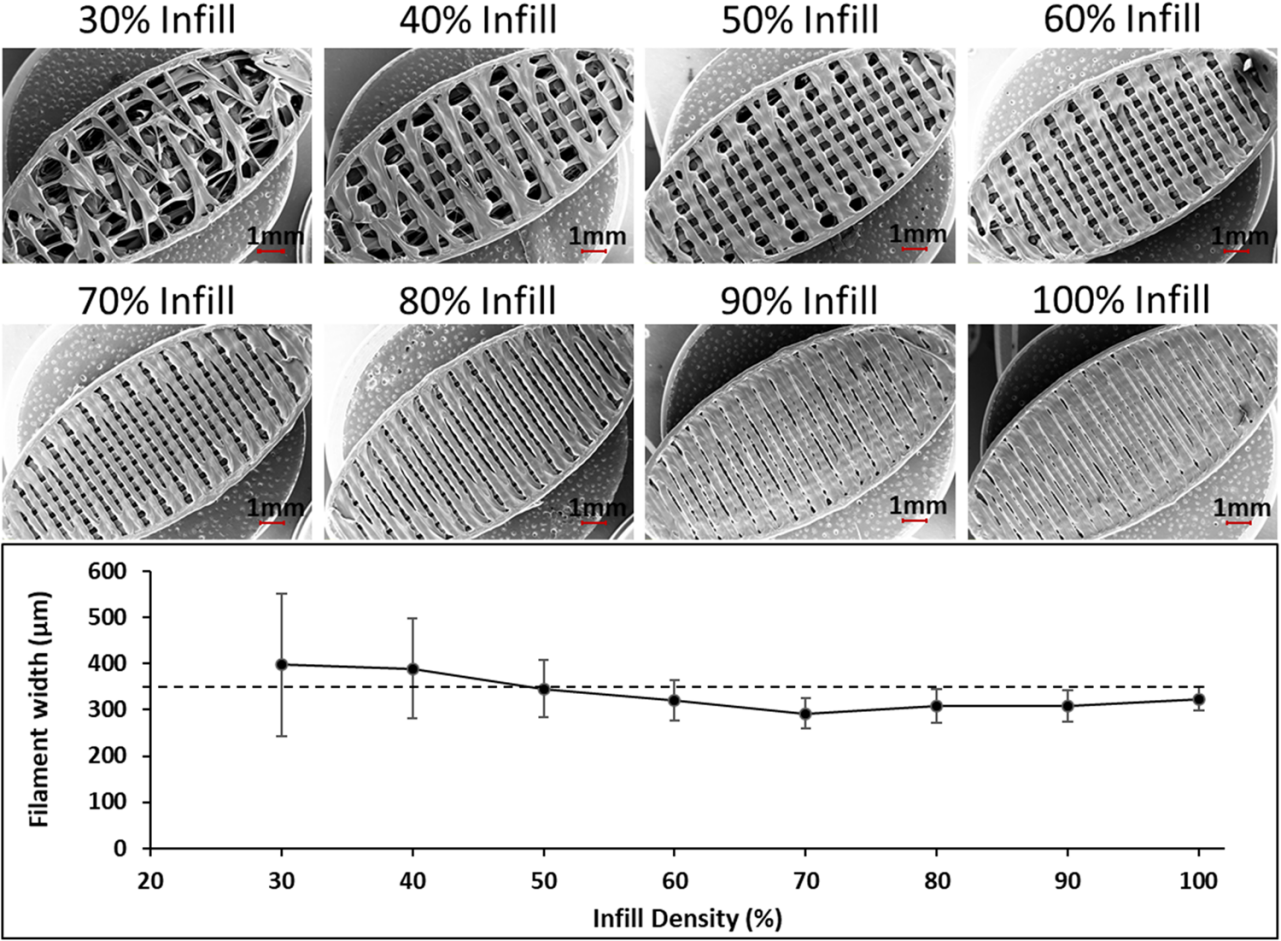
SEM images of APF printed tablets with increasing infill density (the top surfaces of the tablets) (top); (b) microscopic printpath dimensions measured from the SEM images (bottom)

When the microscopic morphology of the APF printed tablets were inspected using SEM, it revealed significant merging and dragging of the printpaths in the tablets with 30 and 40% infill. At the turning points of the printpaths, merging can also be observed in all tablets. This is likely to be caused by the combination of the high viscosity of the material and the rapid change of nozzle speed at the turning points of the printpath. When the widths of individual printpaths were analysed (Fig. 6 bottom), high variations are seen in the tablets with 30–50% infills. Printpath uniformity can be seen to improve with infill density, with tablets between 60-100% infill showing low variability. In the literature, the microscopic printing quality, i.e. the uniformity of the printpath width, is rarely analysed and reported [42–44]. Therefore, it is difficult to directly compare the microscopic printing quality of APF tablets with the tablets printed using FDM. In addition, the printing quality can be highly material dependent. In our case, APF printing with the direct granulation feeding enabled the use of a single polymeric excipient with no addition of plasticiser. This is impossible to achieve with many polymers using FDM printing.

**Fig. 7:**
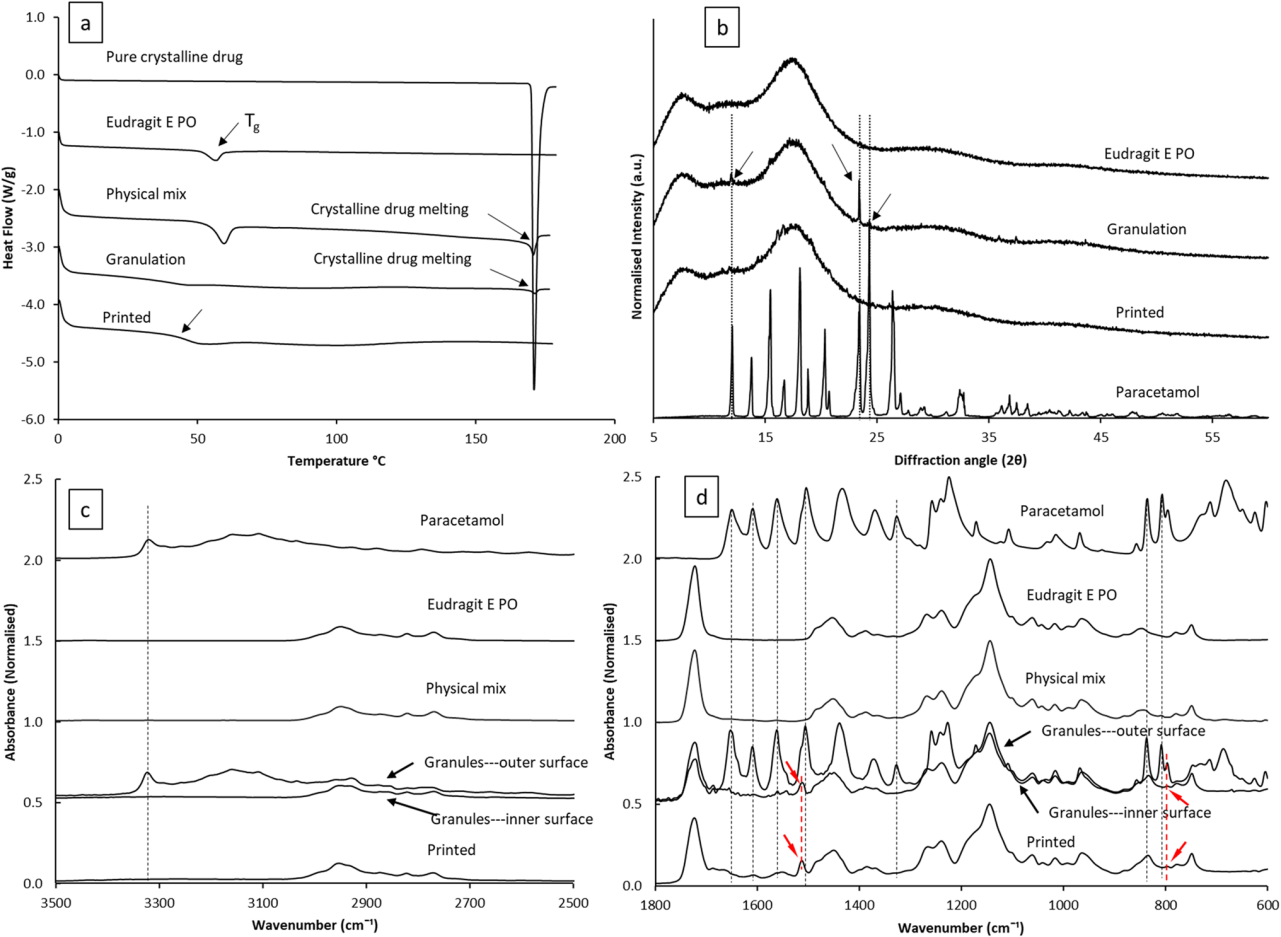
**a** DSC thermogram and **b** PXRD data and **c** partial ATR-FTIR (3700–2500 cm^−1^) and **d** partial ATR-FTIR (1800–600 cm^−1^) of raw materials, physical mixes, granules and APF printed tablets

Thermal transitions for the raw materials were measured using DSC shown in Fig. 7a. The melting peak of paracetamol occurred at 170 °C and the T_g_ of Eudragit was identified as 56.6 °C, agreeing with reported values in the literature [45, 46]. The enthalpy value of the crystalline drug melting reduced significantly in the DSC result of the granules in comparison to the physical mixes. This indicates a certain proportion of the paracetamol dissolved during the granulation process. This was further confirmed by the shift of the T_g_ of granulated materials. The T_g_ of the granulated materials is detected at 41.6 °C. This reduction is due to the plasticisation of dissolved paracetamol. No crystalline drug melting was detected in the APF printed tablet samples. The measured T_g_ of the APF printed tablets is 43.5 °C. Using the Gordon-Taylor (G-T) calculation [47], the predicted T_g_ of a fully amorphous molecular dispersion of paracetamol in Eudragit with a ratio of 1:10 (w/w) is 51.9 °C. As shifts from G-T predicted T_g_ are common for solid dispersions and can be caused by drug-polymer interactions, it is reasonable to suggest that the paracetamol was fully incorporated in Eudragit as a molecular dispersion in the printed tablets.

PXRD data was used to further confirm the physical states of the drug in the granules and the printed tablets. As the drug loading is 9.1% w/w, the diffraction pattern of the granules is largely dominated by the amorphous nature of the polymer (Fig. 7b). However, there was an indication of crystallinity in the granules with two peaks visible at 12.0° 2θ and 23.4° 2θ. These peaks align well with the peaks identified in the diffraction pattern of the crystalline paracetamol form I, indicating no polymorphic change caused during the granulation process. In the diffraction pattern of the APF printed tablets, these peaks have disappeared suggesting paracetamol is mainly amorphous within the printed tablets agreeing with the FTIR data.

ATR-FTIR was further used to examine the physical state of the model drug in the granules and the APF printed tablets. The physical mixture showed low intensity of the peaks associated with paracetamol, most likely due to the heterogeneous sampling of the powder mixtures. ATR-FTIR is able to distinguish between surface and bulk signals since the penetration depth of the light into the sample is of the order of 1 µm. Sampling the whole granule therefore gives information about the surface of the material and sampling a cross section gives information about the bulk. In this particular case, large granules were used and sectioned to reveal the inner cross-section. As seen in Fig. 7c and d , the ATR-FTIR spectra of the outer and inner surfaces are significantly different. The spectra of outer surfaces of the granules contain all the main peaks associated with the presence of crystalline paracetamol. The peaks associated with characteristic vibrational peaks of crystalline paracetamol are N–H stretching at 3315 cm^−1^, Amide I and C = C stretching at 1651, and 1608 cm^−1^, respectively, amide II at 1562 cm^−1^, asymmetrical C-H bending at 1506 cm^−1^ and C–C stretching appeared at 1433 cm^−1^, symmetrical C-H bending at 1369–1329, and para-disubstituted aromatic ring and out of plane ring deformation of phenyl ring, at 837 and 808 cm^−1^, respectively. However, most of these peaks were absent in the spectra of the inner surfaces of the granules and only a few peaks associated with amorphous paracetamol are visible, including the peaks at 798 and 1508 cm^−1^. This result indicates paracetamol is crystalline on the outer surfaces of the granules and amorphous inside the granules. This is consistent with the PXRD and DSC data which show a small amount of crystalline material together with amorphous material. These observations may be attributed to the drying process of the granules. Water was used as the granulating fluid in this study which could dissolve a certain proportion of the crystalline paracetamol, but not Eudragit. During drying of the granules, water containing dissolved paracetamol migrated to the surface prior to being evaporated, leaving paracetamol to concentrateon the outer surface of the granules and recrystallise. The spectra of the printed tablets are very similar to the ones of the inner surfaces of the granules, in which only the presence of amorphous paracetamol can be identified. This is in good agreement with the DSC results and literature data on similar solid dispersions containing amorphous paracetamol, confirming the drug is in an amorphous state in the printed tablets [48, 49]. Considering the printing temperature used in APF printing is between 140–155 °C (which is below the melting point of crystalline form I paracetamol), we believe that the fully amorphous nature of the drug in the printed tablets is due to the well documented paracetamol melting point depression caused by thermal mixing with Eudragit® E PO [45, 48].

### Drug content

Drug content assays of printed material were measured at 99.3% ± 2.0 of the theoretical drug load (9.1% w/w) indicating no significant thermal degradation or change to API loading following granulation and printing. Results were compared to existing pharmacopeia regulations for batch produced tablets as a guide for assessing the accuracy and reliability requirements necessary for pharmaceutical solid dosage form approval. The European Pharmacopoeia test for uniformity of content (Ph. Eur. (2.9.6) [50] is a test intended for solid dosage forms that are batch produced in large quantities and requires the drug contents of tested samples (n = 20) to be within 85–115% of the average value. The drug content uniformity results of the APF printed tablets were within 96.6–102.2% of the mean drug content (based on data from 6 replicates), demonstrating content uniformities well within these requirements. We recognise that the sampling of the tablets here reported does not conform to current pharmacopeia regulations which are predicated on large scale production of tablets. 3D printing is proposed as a route to personalised medicine that will produce small batches of tailored dosage forms and will probably require new accreditation methods. We do not seek therefore to conform to the European Pharmacopoeia test for uniformity of content (Ph. Eur. (2.9.6) but to show the potential of the APF method to meet the requirement that drug contents of all tested samples be consistent with current European Pharmacopoeia requirements.

### *In vitro* drug release

**Fig. 8:**
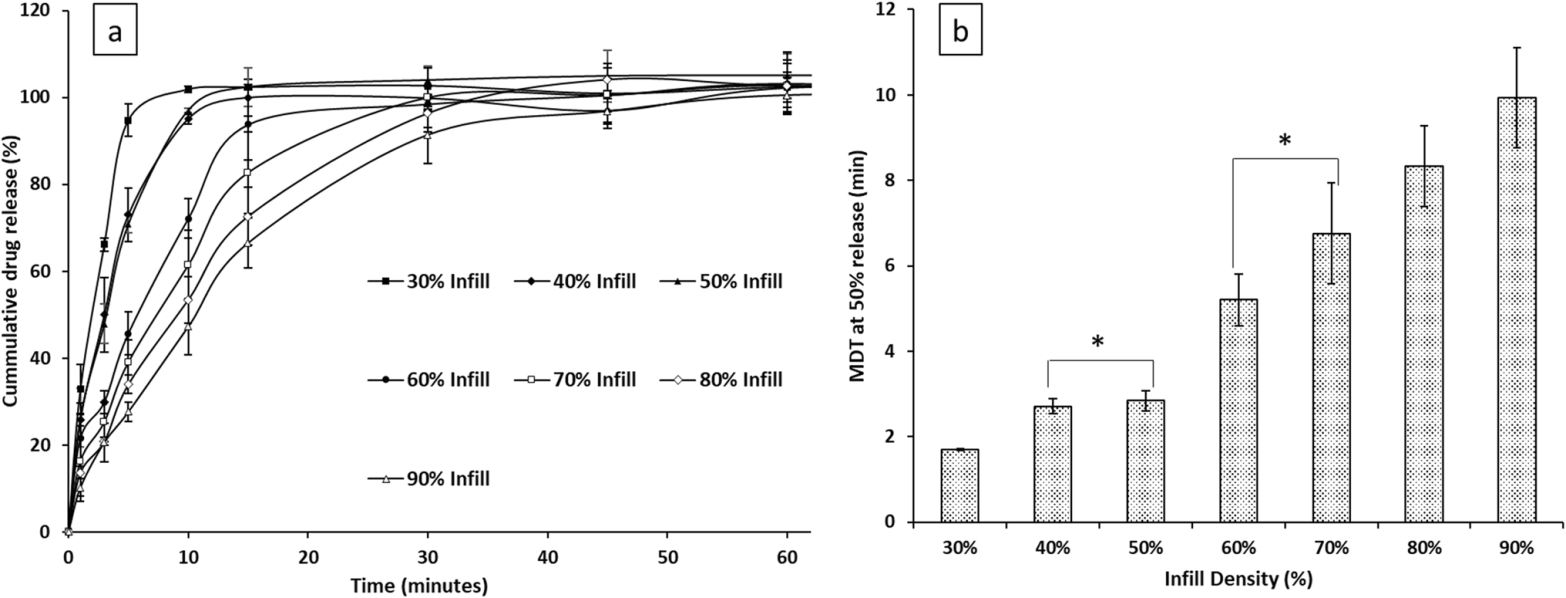
**a** In vitro dissolution data of the APF printed tablets with infills from 30 to 90% in pH 1.2 HCl. **b** Mean dissolution time of the tablets with different infills. The mean dissolution times of the tablets with 40 and 50% infill are not statistically significantly different. Same insignificance was observed between the MDT of the tablets with 60 and 70% infills. (* indicating insignificant difference)

The in vitro drug release data of the APF printed tablets with different infills are shown in Fig. 8a. As infill percentage decreases the SA/V ratio increase resulting in faster release rates [51, 52]. As Eudragit is soluble at pH 1.2 and paracetamol is highly soluble, even the 90% infill tablet reached > 90% drug release within 30 min. For immediate release dosage forms Pharmacopeia standards require at least 80% of the active substance to be dissolved within 45 min (Ph. Eur. (5.17.1)) [53]. All the tablets easily satisfied these requirements indicating the suitability of Eudragit E PO for immediate release applications. With decreasing infill density, release rates were seen to increase further with the 30% infill tablet reaching > 90% drug release in 5 min. It is difficult to conclude directly from the dissolution profiles whether the drug release kinetics of the tablets with different infills are statistically significant from one another. Therefore, the mean dissolution time (MDT) was introduced and calculated. As seen in Fig. 8b, the trend of increasing MDT values with tablet infill was statistically significant but there was no statistically significant difference between the 40 and 50% infill tablets and the 60 and 70% infill tablets. These results confirmed that although infill can be used as a tool for modulating drug release, care should be taken with the increments needed to induce significant change in drug release kinetics.

## Conclusion

This study is the first report on using APF to print solid porous dosage forms via direct granule feeding. The results demonstrated for the first-time direct 3D printing of a brittle pharmaceutical polymer, Eudragit E, without the need for any additives. This new process also bypasses the filament making process and significantly reduces the risk of thermally induced instabilities of the drug and excipients in comparison to conventional filament fusion-based 3D printing. Granulation is a mature pharmaceutical manufacturing process. It allows the compounding of powder from raw materials into granules to avoid the direct handling of powders as an intermediate during 3D printing, which reduces the complexity of the infrastructure requirement for powder handling and the risk associated for operators. The printing quality and performances of the porous tablets were confirmed to be highly compliant with the pharmacopeia requirement. Taking into consideration the large building chamber, multiple printing heads and all sterilisable metal parts of the printers that are in direct contact with the printing materials; APF printing via direct granule feeding has good potential to be adapted and used within cGMP standards and can be a valuable alternative 3D printing method for pharmaceutical solid dosage form manufacturing at/close to the point of care for personalisation.
